# Lateral Penumbra Modelling Based Leaf End Shape Optimization for Multileaf Collimator in Radiotherapy

**DOI:** 10.1155/2016/9515794

**Published:** 2016-01-19

**Authors:** Dong Zhou, Hui Zhang, Peiqing Ye

**Affiliations:** Department of Mechanical Engineering, Tsinghua University, Beijing 100084, China

## Abstract

Lateral penumbra of multileaf collimator plays an important role in radiotherapy treatment planning. Growing evidence has revealed that, for a single-focused multileaf collimator, lateral penumbra width is leaf position dependent and largely attributed to the leaf end shape. In our study, an analytical method for leaf end induced lateral penumbra modelling is formulated using Tangent Secant Theory. Compared with Monte Carlo simulation and ray tracing algorithm, our model serves well the purpose of cost-efficient penumbra evaluation. Leaf ends represented in parametric forms of circular arc, elliptical arc, Bézier curve, and B-spline are implemented. With biobjective function of penumbra mean and variance introduced, genetic algorithm is carried out for approximating the Pareto frontier. Results show that for circular arc leaf end objective function is convex and convergence to optimal solution is guaranteed using gradient based iterative method. It is found that optimal leaf end in the shape of Bézier curve achieves minimal standard deviation, while using B-spline minimum of penumbra mean is obtained. For treatment modalities in clinical application, optimized leaf ends are in close agreement with actual shapes. Taken together, the method that we propose can provide insight into leaf end shape design of multileaf collimator.

## 1. Introduction

Lateral penumbra of single-focused multileaf collimator has been recognized as one of the key dosimetric characteristics and has a significant impact on dose delivery accuracy in radiation therapy treatment planning [1, 2]. Penumbra characteristics strongly influence the amount of healthy tissue involvement, especially where sharp dose gradient is required for stereotactic body radiation therapy.

Penumbra is typically defined as the distance over which the dose profile falls from 80% to 20% [3]. For clarification, penumbra is classified into two categories, namely, fluence penumbra and dosimetric penumbra. Fluence penumbra is acquired on the in-air scoring plane, and it is composed of geometric penumbra and transmission penumbra. Fluence penumbra is also known as in-air penumbra. In contrast, dosimetric penumbra is measured within phantom, and it is the combined effect of fluence penumbra and phantom scatter factor. Dosimetric penumbra is also referred to as physical penumbra or clinical penumbra. In what follows we refer exclusively to fluence penumbra of single-focused multileaf collimator.

Intensive studies have been carried out to explore the experimental penumbral properties for radiotherapy modalities [4, 5]. Results have shown that lateral penumbra is significantly correlated with source model, leaf position, and leaf end shape. On the other hand, the impact of rounded leaf end effect on radiation field offset has been well understood and quantified. Growing evidence has also revealed that penumbra region is field size dependent and largely attributed to the leaf end shape [6, 7].

Researches on leaf end design have been conducted in the past decades [8]. Based on chord intersection assumption, empirical method for leaf end shape optimization has been utilized for obtaining optimal radius of leaf end in the shape of symmetric circular arc. Analytical expressions of geometric penumbra and transmission penumbra have been derived separately. With focal spot size incorporated, computer simulation has shown that leaf end with slightly smaller radius than the empirical result would be suggested. However, problems remain in terms of modelling accuracy of leaf end induced total penumbra and leaf end shape optimization if geometric parameterization is more complex than symmetric circular arc. This begs the question, “How to get physically the lateral fluence penumbra performance by means of leaf end shape optimization?,” which is the motivation for the study.

In terms of total penumbra modelling [9], full Monte Carlo simulation has been proved to be the most accurate among currently available algorithms. However, detailed radiotherapy geometries should be provided in practice and a large number of particle histories have to be recorded in order to achieve desired outcomes. Alternatively, leaf end correction based ray tracing algorithm was found to be easy to implement and could potentially achieve the desired accuracy, which deploys simplified geometries and virtual source models [10]. Since shape optimization process typically involves a large number of penumbra function evaluations in order to search for global optimum, the difficulty of ray tracing algorithm lies in the fact that computation time and penumbra accuracy are largely dependent on discretization error of radiation field and virtual source.

The framework of our work is primarily made up of four parts. In the first part, for the purpose of fast penumbra evaluation, a novel method for leaf end induced penumbra modelling is presented based on Tangent Secant Theory (TST). Compared with Monte Carlo simulation and ray tracing algorithm, TST penumbra modelling is proved to be cost-efficient. In the second part, a variety of leaf end parameterization techniques are investigated. In the third part, leaf end shape design is formulated as a problem of biobjective optimization with mean-variance cost function. Genetic algorithm based global optimization and gradient based local optimization are deployed. In the last part, radiotherapy geometries of Agility 160-leaf MLC (Elekta AB, Stockholm, Sweden) and Millennium 120 MLC (Varian Medical System, Palo Alto, CA, USA) are utilized to testify the feasibility of TST modelling and leaf end shape optimization method.

## 2. Materials and Methods

### 2.1. Tangent Secant Theory for Penumbra Modelling

**Figure 1:**
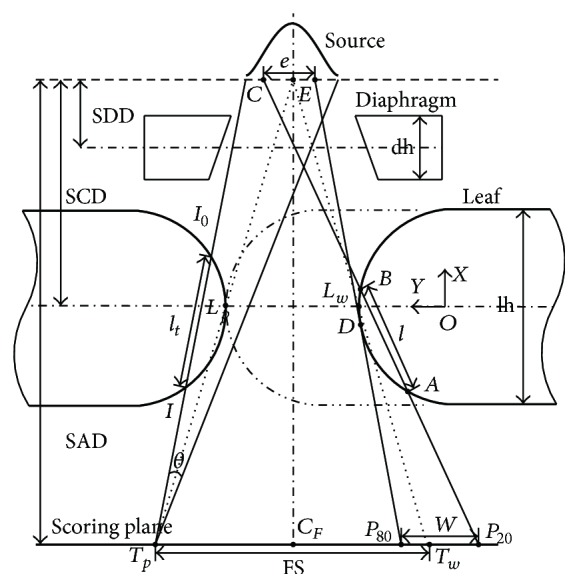
TST penumbra modelling.

As illustrated in Figure 1, given an arbitrary point *T*_*p*_ on the scoring plane, ray tracing algorithm for calculation of beam intensity at *T*_*p*_ is utilized by summation of weighted beam intensity within the angle of *θ*. Considering beam penetrating leaf end with path length *l*_*t*_ and density *ρ*, emerging beam intensity *I* is related to incident beam intensity *I*_0_, which is given by the exponential attenuation law, usually referred to as Beer-Lambert law:

(1) $$\begin{array}{c} I=I_{0}e^{-\left(\mu_{a}/\rho \right)\rho l_{t}}, \end{array}$$

where *μ*_*a*_ is the attenuation coefficient and *μ*_*a*_/*ρ* denotes X-ray mass attenuation coefficient.

In contrast, the main idea of TST penumbra modelling is that, for arbitrary leaf position, penumbra width is obtained by directly searching for two points, the 80% intensity point and the 20% intensity point, where the 100% intensity point is measured at the centre of radiation field *C*_*F*_. Therefore, computational efficiency can be realized, without calculating full-field dose profile.

The modelling method is depicted as follows. Given an arbitrary physical position of leaf end *L*_*w*_, the projection of *L*_*w*_ onto the scoring plane is point *T*_*w*_. Penumbra width *W* is determined by the 80% intensity point *P*_80_ and the 20% intensity point *P*_20_. On the one hand, *P*_80_ is approximated by visible source area integration method, which is utilized by projecting multileaf collimator back to the source plane and summing up the visible area of source profile. Firstly, cumulative distribution function of source profile is expressed as the integral of its probability density function, and the 80% cumulative intensity on source profile is obtained by one-dimension search technique, which denotes point *E* in Figure 1. Secondly, by drawing tangent line of leaf end from point *E*, *P*_80_ on the scoring plane is obtained. On the other side, point *P*_20_ is obtained by drawing a secant line from source point *C* to the scoring plane, with path length defined by chord *AB* on leaf end curve. *C* is defined by the equivalent source size length *e* and *AB* is defined by the effective path length *l*. As a consequence, TST penumbra modelling employs only two variables, namely, *e* and *l*.

Based on mathematical optimization, iterative approaches for deriving the tangent line and secant line are introduced. For details, refer to Appendices A and B. Intercept theorem is used for penumbra evaluation, and the relations are written with the notation:

(2) Ws,v=yP80−yP20,yP80=yE−yDxE−xDxCF−xD+yD,yP20=yC−yBxC−xBxCF−xB+yB,s=e,l,v=Cp,T,

where the origin of the coordinate system is placed at the centre of circular arc. *D* denotes point of tangency. Penumbra *W* is the function of system related vector **s** and leaf end related vector **v**. Vector **s** is composed of the equivalent source size and the effective path length, and vector **v** is composed of leaf end curve **C** and leaf end position **T**. Leaf end curve **C** is determined by design variables **p**. For circular arc leaf end, design variables include arc radius and centre offset.

The equivalent source size and the effective path length are obtained by parameter identification, which involves two steps. Firstly, with curves grouped according to design variables, reference data of leaf position related penumbra are obtained by Monte Carlo simulation or ray tracing algorithm. Secondly, nonlinear least squares based curve fitting is introduced to determine the equivalent source size and the effective path length. Mathematical term is as follows:

(3) mine,lWs,v−W−C,T2=mine,l∑i∑jWi,je,l,Ci,Tj−W−i,jCi,Tj2,

where **W** and $\overset{-}{W}$ denote penumbra matrices of TST model results and reference data, respectively. *W*_*i*,*j*_ denotes the element of TST penumbra matrix. ${\overset{-}{W}}_{i,j}$ denotes the element of reference penumbra matrix, which is obtained by Monte Carlo simulation or ray tracing algorithm. **C**_*i*_ denotes the leaf end curve *i*, and *T*_*j*_ denotes the leaf position *j*.

### 2.2. Leaf End Shape Parameterization

Topolnjak and van der Heide [8] suggested leaf end designed in the shape of elliptical arc could be beneficial for penumbral properties. Intuitively, polynomial curves are superior in terms of the ability to handle local shape changes and the availability to obtain sensitivity derivatives. Therefore, apart from circular arc leaf end, the potentials of elliptical arc, Bézier curve, and B-spline are explored and rigorously investigated in our work.

**Figure 2:**
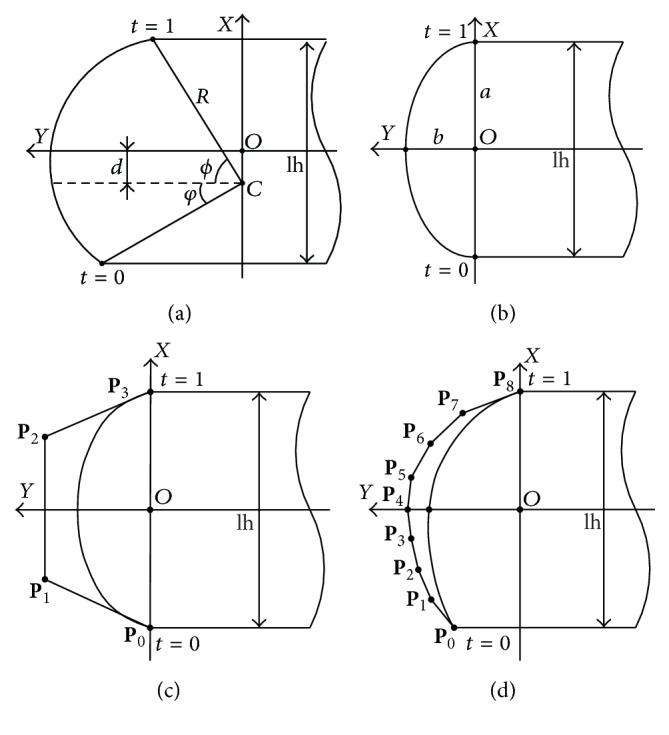
Leaf end shape parameterization: (a) circular arc, (b) elliptical arc, (c) Bézier curve, and (d) B-spline.

Note that the path length of beam penetration through leaf entity is related to the distal and proximal leaf edge. A piecewise parametric leaf curve is established, which consists of three segments, the leaf end curve, the distal leaf edge, and the proximal leaf edge. Origin of the coordinate system is placed on the leaf average height, and the longitudinal axis *y* is in accordance with leaf motion. Let lh denote leaf height; leaf end shape parameterization is depicted in Figure 2.

Circular arc and elliptical arc are regularized as parametric curves. The uniform representation of leaf curves is as follows:

(4) $$\begin{array}{c} C=\left\{\;\left(\;x\left(\;p,t\;\right),y\left(\;p,t\;\right)\;\right)\;|\;t\in \left(\;-\infty ,+\infty \;\right)\;\right\}, \end{array}$$

where **C** is the set of points that satisfy leaf curve parametric equations. Point on leaf curve with design variables **p** is a function of independent parameter *t*. For *t* < 0 and *t* > 1, parametric curve point is on the distal and proximal leaf edge. Mathematical terms are given as follows:

(5) x=−lh2,y=t+yp,0,t∈−∞,0,x=lh2,y=1−t+yp,1,t∈1,+∞.

For *t* ∈ [0,1], four kinds of leaf end curve parameterization techniques and their design variables are listed in Table 1.

### 2.3. Verification and Validation of TST Penumbra Model

Verification and validation of TST penumbra model involved three steps. Firstly, leaf position-penumbra curves grouped by radius of circular arc leaf end are obtained by Monte Carlo simulation. Secondly, the equivalent source size and the effective path length are obtained using parameter identification, with reference penumbra data obtained from Monte Carlo simulation. Thirdly, with Gaussian source approximation, results of ray tracing algorithm are presented for comparison.

Numerical simulation is based on EGSnrc/BEAMnrc Monte Carlo codes [11]. In our study, monoenergetic source of 1.5 MeV is adopted, which is the average energy for 6 MeV source. Source size with Gaussian distribution of 0.2 cm full width at half maximum (FWHM) is used. Leaf is made of W700ICRU, with density *ρ* of 19.3 g/cm^3^. X-ray mass attenuation coefficient *μ*_*a*_/*ρ* of tungsten leaf is 0.05 cm^2^/g for photon energy of 1.5 MeV [12]. Beam angle *α*_*B*_ about collimator rotation axis is 15.8°, which is determined by field size (FS) and source to axis distance (SAD).

In order to estimate the contribution of leaf end shape to penumbral properties, we develop a specific geometric model for verification and validation of TST model. Parameters of geometric model are listed in Table 2. In order to obtain leaf position-penumbra curve, field size shaped by leaf ends is designated as 10 × 10 cm^2^, which remains unchanged while the centre of the radiation field shifts.

Virtual energy fluence source models, including single source model, dual source model, three-source model, and hybrid source model, have been proved to be feasible for photon source modelling [13]. By substitution of Gaussian source with virtual source models, various source distributions could be made possible in our program.

### 2.4. Leaf End Shape Optimization

#### 2.4.1. Penumbra Mean-Variance Optimization Objectives

Idealized collimator system for clinicians should meet the requirements of minimal penumbra width and consistent field variance [8]. To this end, leaf end shape design is formulated as a biobjective problem of penumbra mean-variance optimization, which attempts to minimize penumbra mean for a given penumbra variance or equivalently minimize penumbra variance for a given level of penumbra mean, by carefully choosing leaf end shape. It can be written in the following notation:

(6) min Jp=J1p,J2p,w.r.t.  ps.t. bl≤p≤bu Ap≤bg Qkp≤0,k=0,1,…,lnwhere J1p=μWs,v J2p=σWs,v s=e,l, v=Cp,T,

where **J** is the biobjective function, *J*_1_ denotes penumbra mean *μ*, and *J*_2_ denotes standard deviation *σ*. Constraint functions include bound constraints **b**_*l*_, **b**_*u*_, linear constraints **b**_*g*_, and nonlinear constraints *Q*(**p**). For details, refer to Table 3.

In our study, leaf position-penumbra curve derivation is composed of two steps, namely, field discretization and sample point evaluation. Let *T*_*j*_ be leaf position on the scoring plane. Penumbra mean and standard deviation are expressed mathematically as follows:

(7) μW=1N∑j=1NWjs,v,σW=1N−1∑j=1NWjs,v−μW2,Wjs,v=Wje,l,Cp,Tj,yTj=yTw+j−1N−1yTp−yTw,j=1,…,N,

where *T*_*p*_ is the leaf end point on scoring plane when leaf protrudes fully across the central axis. Point *T*_*w*_ is denoted by the position where leaf is fully withdrawn. Distance between *T*_*p*_ and *T*_*w*_ denotes leaf stroke. Uniform sampling method is adopted to obtain leaf position *T*_*j*_. On account of mechanical constraints, leaf stroke can be asymmetric about the collimator rotation axis.

#### 2.4.2. Convex Hull Assumption of Leaf End and Optimization Constraints

It is stated that for a given concave leaf end the corresponding convex hull leaf end can achieve better penumbra performance. A brief proof is presented for illustration. Based on computational geometry theory, convex hull leaf end is designated as the intersection of all sets of convex leaf ends containing the concave leaf end. Note that the relative complement of the convex hull leaf end with respect to the concave leaf end is filled with leaf material for the convex hull leaf end. This observation implies that radiation beams require extra path length for penetration through the convex hull leaf end. Consequently, radiation beams attenuate more quickly and sharper radiation field edge should be achieved. Under the convex hull assumption and geometry boundaries, leaf curve constraints are listed in Table 3. For simplicity, cubic Bézier curve is used with fixed starting point and ending point. Cubic B-spline with 9 control points is utilized with ending point fixed and degree of freedom along *y*-axis constrained.

#### 2.4.3. Multiobjective Optimization and Pareto Frontier Approximation

Considering that objective space is convex for circular arc and elliptical arc, gradient based iterative algorithm is robust and efficient for shape optimization. Since shape optimization typically involves multiple control variables for Bézier curve and B-spline, it is difficult to distinguish the potential global optimum from local optimum without function convexity information provided in advance. In view of multiple local optima which exist in the objective space, genetic algorithm based global optimization is implemented for approximating Pareto frontier without being trapped near local optima.

A weighted sum approach is introduced by creating a scalar function for mean-variance biobjective shape optimization. A tuning coefficient is introduced to modulate the weight between penumbra mean and standard deviation. In mathematical terms, it can be formulated as

(8) $$\begin{array}{c} \min J_{tot}=\lambda \mu +\left(\;1-\lambda \;\right)\sigma ,\;\lambda \in \left[0,1\right], \end{array}$$

where *J*_tot_ denotes the composite objective function. By systematically changing the weight among objectives, the Pareto frontier is obtained. Since function evaluation is most time-consuming, parallel computing technique is used to speed up the process.

### 2.5. Implementation of Leaf End Shape Optimization

Matlab codes (Mathworks, Natick, MA, USA) have been developed to compute the leaf position-penumbra curve and solve the problem of leaf end shape optimization. Besides the proposed geometric model, the method that we propose is utilized with treatment modalities of Elekta Agility 160-leaf MLC and Varian Millennium 120 MLC. Comparison is conducted with empirical method [8]. The optimal radius equation using empirical method is written as

(9) $$\begin{array}{c} R_{opt}=\frac{1}{2}{\left(\;a_{1}^{2}+4a_{1}^{2}a_{2}^{2}+4a_{1}a_{2}^{2}a_{3}a_{4}+a_{4}^{2}+4a_{2}^{2}a_{4}^{2}\;\right)}^{1/2}, \end{array}$$

where

(10)   a1=ln0.2μa,a2=SADFS,a3=4·SAD2+FS21/2·SAD−1,a4=lh,

where optimal radius is a function of *μ*_*a*_, SAD, FS, and lh. It is noted that source energy distribution and source to collimator distance are not included in the equation.

## 3. Results

### 3.1. Algorithmic Efficiency

As listed in Table 4, efficiency of three algorithms is investigated for the geometric model, including Monte Carlo simulation, ray tracing algorithm, and TST penumbra model. Leaf position-penumbra curve is obtained using 17 sample points, which are evenly spaced from −20 to 20 cm, with interval length of 2.5 cm. In terms of ray tracing algorithm, three-sigma rule is used to determine the range of source energy distribution. Results have shown that the subsource number of 100 results in source energy error of 1%. Method of reduced space searching is implemented for field discretization, which is implemented by searching only the neighbourhood of leaf end projection point on scoring plane for the 80% and 20% intensity points. It takes 100 segments of truncated penumbra region to achieve calculation error less than 1%.

### 3.2. Verification and Validation of TST Penumbra Model

**Figure 3:**
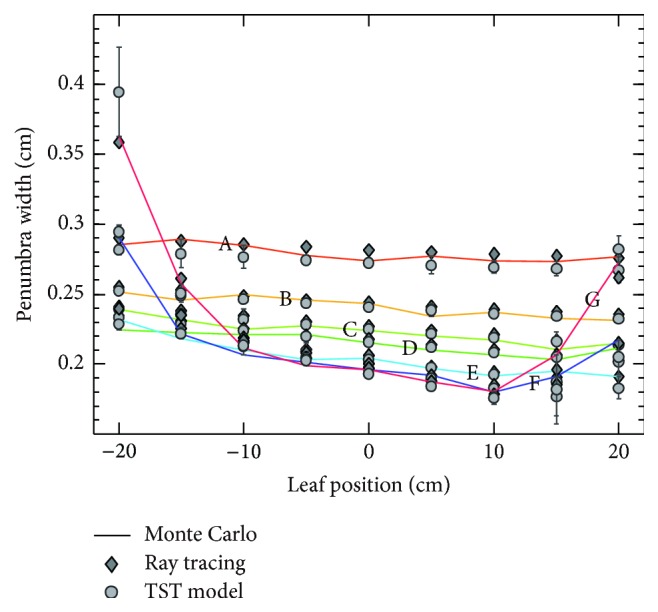
Verification and validation of TST model.

As illustrated in Figure 3, symmetric circular arcs with radius values of 4, 6, 8, 10, 15, 20, and 25 cm are used, which correspond to curves A to G. Error bars indicate the absolute discrepancy between TST model and Monte Carlo simulation. Results of source approximation have shown that penumbra curves of ray tracing algorithm using Gaussian distribution with FWHM of 0.217 cm agree well with Monte Carlo simulation. Parameter identification for TST model reveals that the equivalent source size of TST model is 0.152 cm, and the effective path length is 1.174 cm. Results demonstrate that leaf position-penumbra curves of TST model have a good approximation to Monte Carlo simulation, with maximum value of absolute error 11.9% on curve G. This observation is probably related to the leaf position offset caused by rounded leaf end effect. The observation that curve F and curve G are bowl-shaped is related to beam penetration through the distal and proximal leaf edge.

### 3.3. Circular Arc Leaf End Shape Optimization

**Figure 4:**
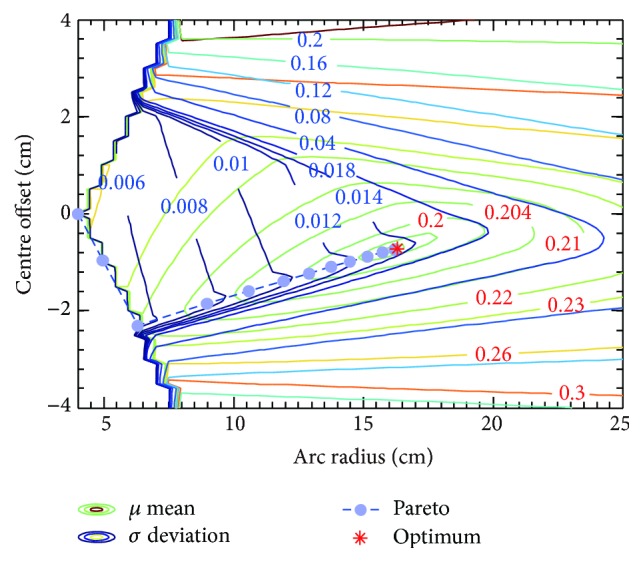
Penumbra mean and standard deviation of circular arc leaf end. Pareto frontier is derived by weighted sum method, with *λ* ranging from 0 to 1. The optimum denotes global minimum of penumbra mean.

Figure 4 shows that penumbra mean contour profile is convex in objective space. Therefore, gradient based optimization algorithm is robust and efficient to search for the global optimum of penumbra mean, and solution is not sensitive to initial guess. For initial guess at radius of 4 cm without centre offset, it takes 53 iterations and 316 function evaluations to converge to global optimum of penumbra mean at radius of 16.213 cm and centre offset of −0.732 cm. On the other hand, the global optimum of standard deviation is at radius of 4 cm without centre offset, which is the minimal radius for leaf height of 8 cm. Note that marginal regions with saw-tooth-shaped edges in Figure 4 are related to constraints on radius and centre offset. The saw-tooth-shaped edge can be eliminated by decreasing interval length of radius and centre offset. In our study, interval lengths are 1 cm and 0.1 cm for radius and centre offset, respectively.

### 3.4. Elliptical Arc Leaf End Shape Optimization

**Figure 5:**
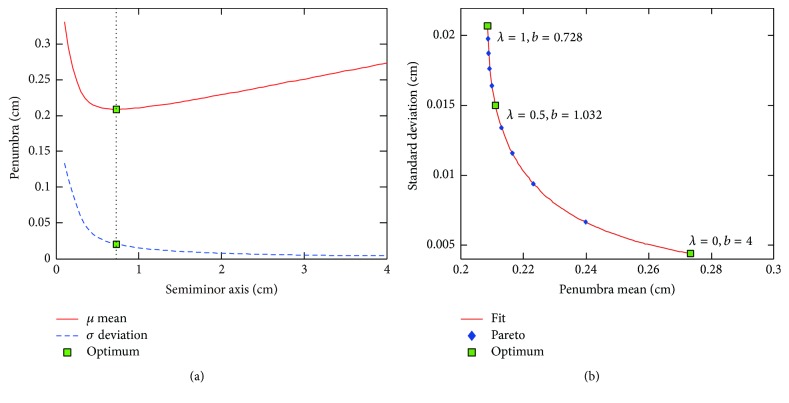
Elliptical arc leaf end penumbra mean and standard deviation: (a) semiminor axis dependent penumbral properties, (b) Pareto frontier of elliptical arc leaf end.

Figure 5(a) demonstrates penumbral properties of elliptical arc leaf end. Notably, global optimum of penumbra mean is reached with semiminor axis of 0.728 cm. Global minimum of standard deviation is reached with semiminor axis of 4 cm, which means leaf end is in the shape of circular arc. Pareto frontier of elliptical leaf end is depicted in Figure 5(b). For *λ* greater than 0.5, penumbra mean is almost invariable, while standard deviation changes rapidly.

### 3.5. Pareto Frontier of Bézier and B-Spline Curves and A Priori Method for Optimal Point Selection

**Figure 6:**
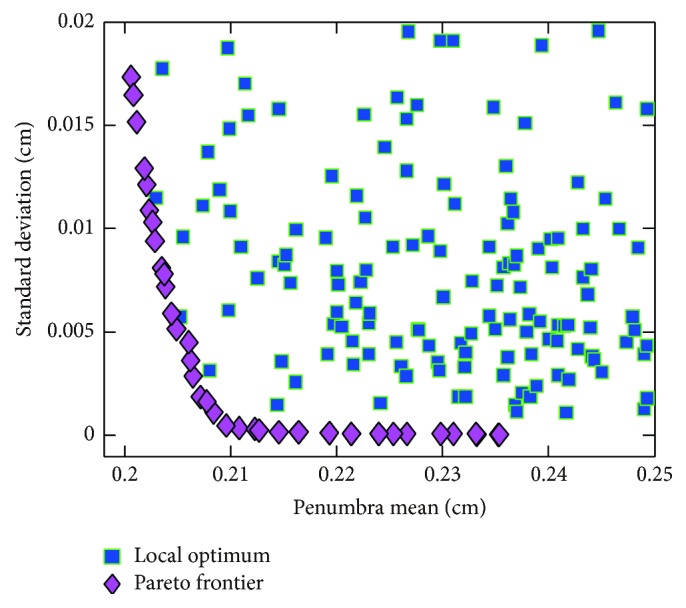
Pareto frontier of cubic Bézier curve. Local optima are obtained by multistart algorithm.

**Figure 7:**
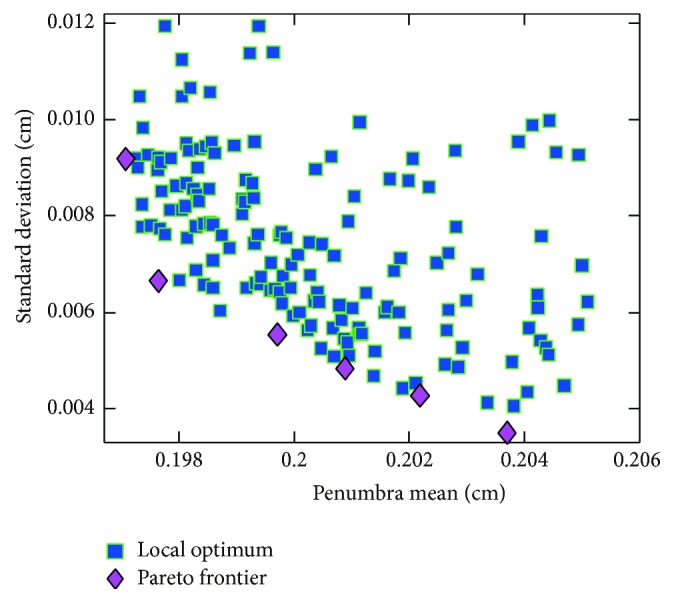
Pareto frontier of B-spline leaf end. Local optima are obtained by multistart algorithm.

Points on Pareto frontier are superior in penumbra mean and standard deviation. As illustrated in Figures 6 and 7, Pareto frontiers of Bézier curve and B-spline are obtained using multiobjective genetic algorithm. Local optimum of penumbra mean is obtained by multistart global optimization algorithm. The data suggest that local optima are spread out over the entire objective space. Therefore, gradient based optimization algorithm converges slowly and gets easily trapped in local optimum. Since penumbra width is of interest for clinical application, it is suggested that penumbra mean is preferred over standard deviation for optimal point selection, which is used as a priori knowledge. Consequently, the search for potential of penumbra mean comes first. After that, searching for point on Pareto frontier with relatively small standard deviation is conducted. Note that the Pareto frontier is V-shaped curve; points located at the near-vertex region are selected in our study.

### 3.6. Optimal Leaf End Shapes and Leaf Position-Penumbra Curves

**Figure 8:**
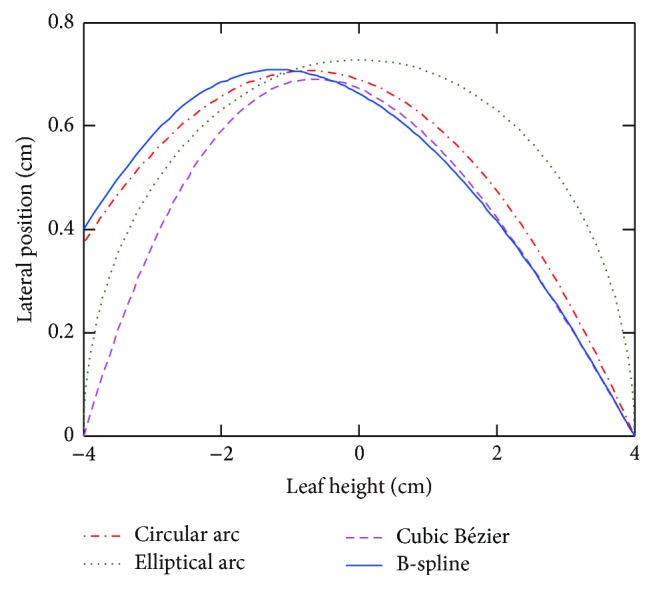
Shape comparison of optimal leaf ends with four parameterization techniques.

**Figure 9:**
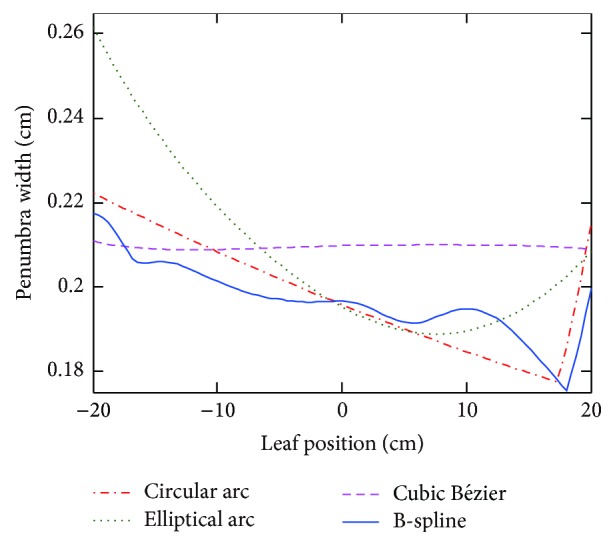
Leaf position-penumbra curves of optimal leaf ends with four parameterization techniques.

Figure 8 compares the shapes of optimal leaf ends using four parameterization techniques. It is noted that optimal leaf ends of circular arc and B-spline resemble each other closely. Leaf position-penumbra curves are depicted in Figure 9. The smooth curve of Bézier leaf end suggests that constant penumbra width can be achieved while maintaining penumbra mean at an acceptable level. B-spline is advantageous in local shape control; thus penumbra mean and standard deviation can be obtained simultaneously. Notably, leaf position-penumbra curve of circular arc is not monotonically decreasing. The observation is related to the fact of beam penetration through the distal leaf edge.

Table 5 summarizes all the optimized leaf ends. Results have shown that penumbra mean values are very close, while standard deviation values vary. Minimum of standard deviation is achieved using Bézier curve, and minimum of penumbra mean is obtained using B-spline.

### 3.7. Combined Effect of SCD and Source Size on Optimal Circular Arc Leaf End

**Figure 10:**
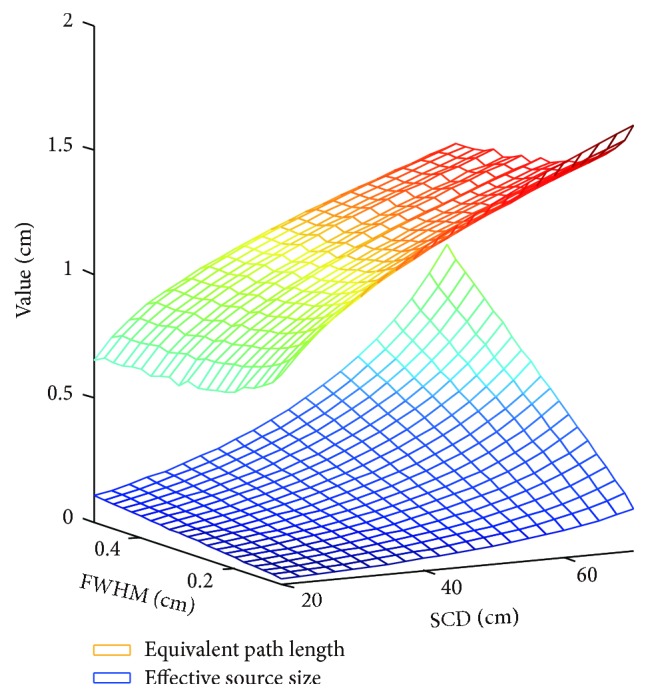
Parameter identification of the equivalent source size and the effective path length based on the geometric model with leaf height of 8 cm.

**Figure 11:**
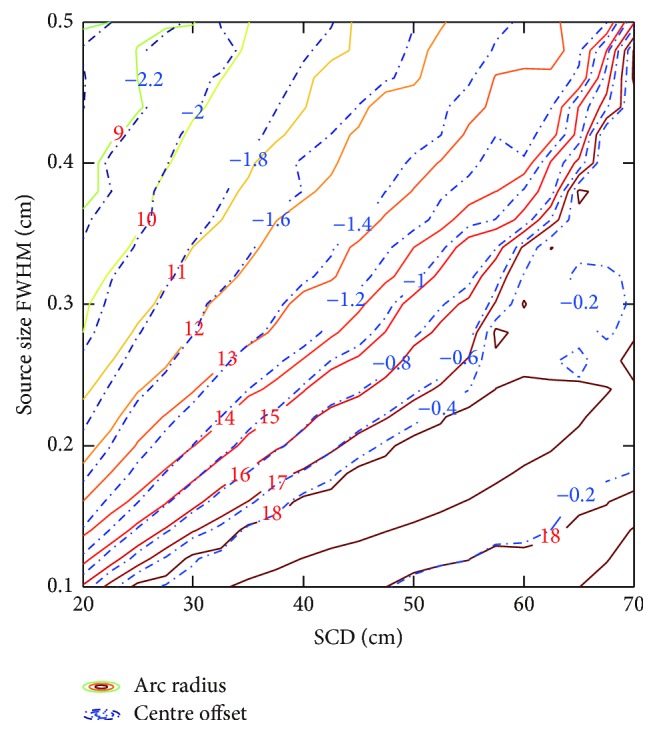
SCD and source size dependent radius and centre offset contour of optimal circular arc leaf ends with leaf height of 8 cm.

As illustrated in Figure 10, combined effect of SCD and source size on the values of the equivalent source size and the effective path length is shown based on the geometric model. Note that a large amount of reference data should be obtained. For the sake of computation time, parameter identification is utilized with the reference data derived from ray tracing algorithm. In Figure 11, results show that optimal circular arc is a function of SCD and source size. Observe that the offset values are uniformly negative. This is mainly related to the effect of geometric penumbra.

### 3.8. Leaf End Shape Optimization for Elekta Agility 160-Leaf MLC and Varian Millennium 120 MLC

**Figure 12:**
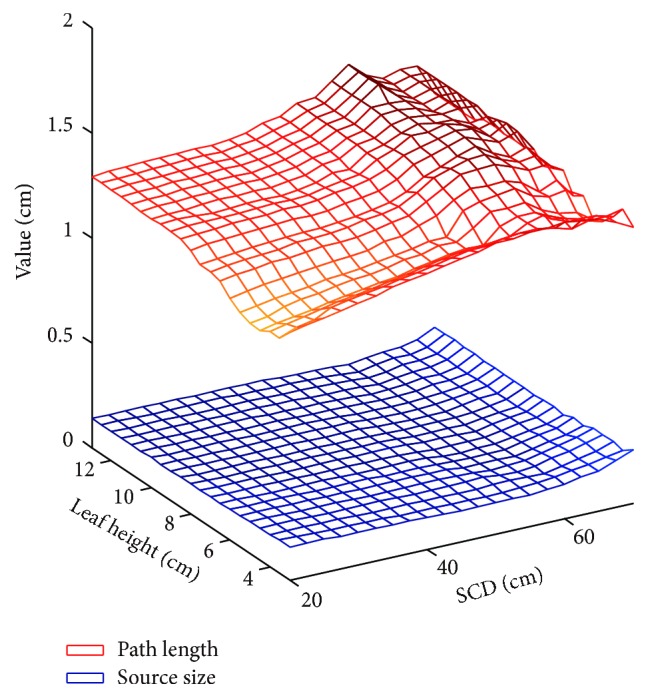
Parameter identification of the equivalent source size and the effective path length.

**Figure 13:**
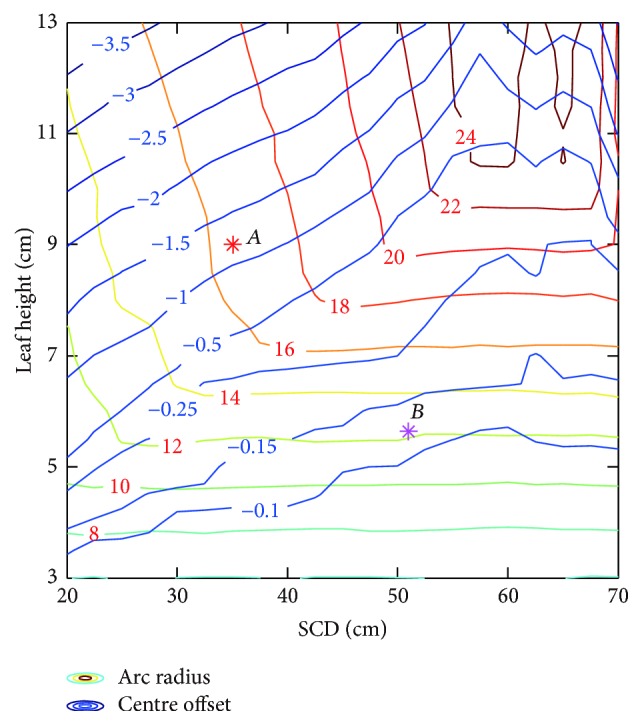
Optimal radius and centre offset of circular arc leaf end.

In our study, Gaussian distribution with FWHM of 0.2 cm is assigned to virtual source. Leaf material is typically heavy tungsten alloy, with density of 18 g/cm^3^. As illustrated in Figure 12, estimation of the equivalent source size and the effective path length is implemented by parameter identification method, with results from ray tracing algorithm for reference.  Figure 13 shows the combined effect of SCD and leaf height on optimal radius and centre offset. Gradient based optimization algorithm is utilized to search for optimal circular arc. Results have shown that the optima are located at point *A* for Elekta Agility 160-leaf MLC and point *B* for Varian Millennium 120 MLC, respectively.

Table 6 summarized the results of optimal leaf ends in the shape of circular arc. Compared with empirical method, TST model can achieve good agreement with actual leaf ends.

**Figure 14:**
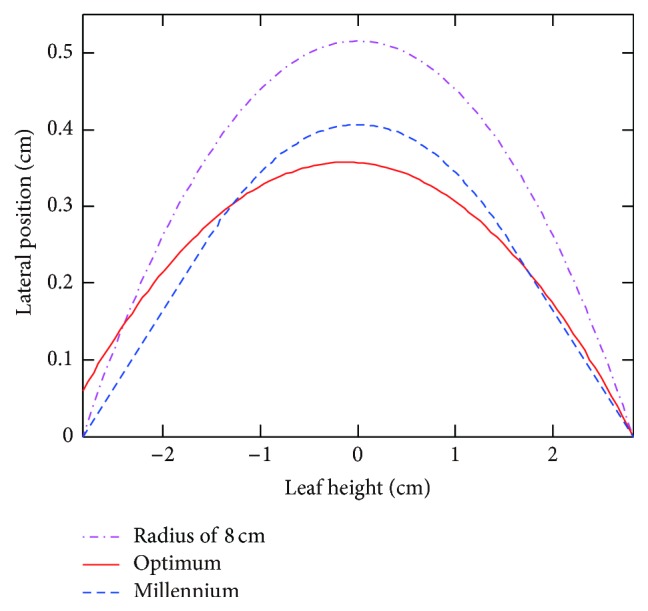
Comparison of optimal circular arc leaf ends using empirical method and TST model with actual shape of Varian Millennium 120 MLC.

Figure 14 illustrates leaf ends of TST result and empirical result, compared with actual leaf end of Varian Millennium 120 MLC. Notably, empirical method results in optimal circular arc radius of 8 cm, while optimal leaf end using TST model is in close approximation to the actual piecewise leaf end, with maximum lateral deviation of 0.06 cm.

## 4. Discussions

Leaf end shape optimization in our study is composed of four steps, geometric model initialization, penumbra evaluation, parameter identification, and Pareto optimization. The results demonstrate that TST penumbra model based mean-variance optimization approach serves well the purpose of leaf end shape design for multileaf collimator in radiotherapy. The results developed herein may be used for leaf end shape design, as well as analytical penumbra calculation. In this study, we employ a specific geometric model, whose parameters are reasonable for multileaf collimator based radiotherapy. Modification can be introduced to adapt to different treatment modalities. These findings will be testified by experimental measurements, in progress in our research group.

Results of optimal elliptical arc manifest the fact that decreasing the semiminor axis value to be less than global minimum of penumbra mean leads to penumbra mean and standard deviation increase sharply. This is due to the edge effect, which is caused by penetration of radiation beams through the proximal and distal leaf edge. Results of optimal Bézier curve indicate that the goal of consistent penumbra variance across the radiation field can be attained; while using B-spline, minimal penumbra mean is made possible. Due to the flexibility of local shape control, B-spline curve with more design variables would be applied to explore the full potential of leaf end shape induced lateral penumbral properties. Besides, piecewise leaf end composed of line segments and curve segments can be introduced. In addition, the stroke length of leaf travel is designated to be symmetric about the collimator rotation axis. However, asymmetric leaf travel range could be implemented.

In principle, choice of the optimal point on Pareto frontier varies according to preference for penumbra mean and standard deviation. In our study, the selection criterion of optimal point on Pareto frontier is based on a priori rule of penumbra mean first. Nevertheless, adjacent points of optimal point could be used with comparable penumbral characteristics and similar leaf end shape.

Objectives of leaf end shape optimization include, but are not limited to, penumbra mean and standard deviation. For better control of leaf position-penumbra curve, we propose penumbra off axis ratio as one of the objective functions, which is expressed arithmetically as the ratio of peripheral penumbra to axis penumbra. Taking clinical practice into account, penumbra located in the neighbouring region of collimator rotation axis is more frequently used. Therefore, a discrepancy of dosimetric effect exists between axis penumbra and peripheral penumbra. In order to account for leaf position dependent dosimetric effect, variant leaf position sampling or weighted penumbra would be introduced for future studies.

Ray tracing penumbra calculation can yield quantitative and accurate results only when discretization error is sufficiently small. By virtue of the development of computation capacity and algorithm improvement, both ray tracing algorithm and Monte Carlo simulation can be accelerated on GPU. For further investigation, ray tracing based leaf end shape optimization or Monte Carlo simulation based optimization would be made possible.

Although kinetic energy of photon beam is assigned to be monoenergetic, functional form of photon spectra model could be adopted for future study. We would like to point out that in our study leaf end shape optimization is largely dependent on fluence penumbra modelling. In view of the fact that dosimetric penumbra in percent depth dose profile can be estimated by kernel based dose calculation method, such as pencil beam kernel based superposition and convolution algorithm, leaf end shape optimization based on direct dosimetric penumbra modelling will be the subject of future publications.

## 5. Conclusions

The purpose of this paper is to introduce an analytical method for leaf end shape optimization that can be easily implemented in leaf end design of multileaf collimator. A cost-efficient modelling method of leaf end induced lateral penumbra is proposed based on Tangent Secant Theory, which is verified by Monte Carlo simulation and ray tracing algorithm. Penumbra mean and variance are introduced for biobjective optimization. Leaf end curve parameterization techniques are introduced, including circular arc, elliptical arc, Bézier curve, and B-spline. Observing that objective space is convex for circular arc and elliptical arc, gradient based iterative method is used for local search, while for leaf ends in the shape of Bézier curve and B-spline, objective space is concave and genetic algorithm is applied to explore the full potential of leaf end shape. Results have shown that our method serves well for efficiently deriving optimal radius and centre offset of circular arc. It is found that leaf position-penumbra curve is flat and the goal of consistent penumbra width can be reached using leaf end in the shape of Bézier curve. Results of optimal B-spline leaf end manifest minimum of penumbra mean due to its flexibility of shape representation. Geometries of treatment modalities manufactured by Varian and Elekta are incorporated for TST model based leaf end shape optimization, and it is shown that a relatively close match is found between optimal leaf end and actual shape.

The method that we propose is feasible to estimate leaf position-penumbra curve and suggest optimal leaf end design for a particular treatment modality. Although in this paper geometries of specific treatment modalities are utilized, the conclusions we reach could provide insight into leaf end shape design of multileaf collimator in radiotherapy.

## Figures and Tables

**Figure 15:**
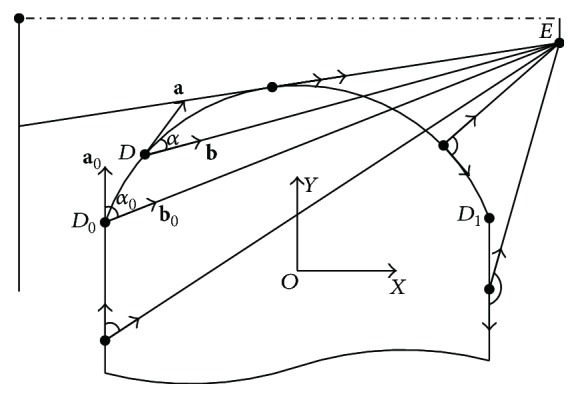
Tangent line algorithm.

**Figure 16:**
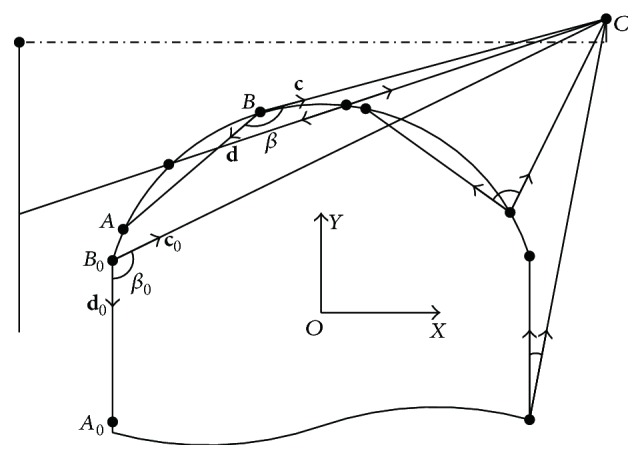
Secant line algorithm.

**Table 1:** Leaf end parameterization and design variables.

Type	Parametric curves	Design variables
Circular arc	$\begin{array}{c} x=-R\cos\left[\frac{\pi }{2}-\varphi +t\left(\phi +\varphi \right)\right]+d\\ y=R\sin\left[\frac{\pi }{2}-\varphi +t\left(\phi +\varphi \right)\right] \end{array}\;\left\{\begin{array}{c} \phi =arcsin\left(\frac{\text{lh}/2-d}{R}\right)\\ \varphi =arcsin\left(\frac{\text{lh}/2+d}{R}\right) \end{array}\right.$	**p** = {*R*, *d*} *R*: radius of circular arc *d*: arc centre offset

Elliptical arc	$\begin{array}{c} x=a\cos\left[(1-t)\pi \right]\\ y=b\sin\left[(1-t)\pi \right] \end{array}$	**p** = {*a*, *b*} *a*: semimajor axis *b*: semiminor axis

Bézier curve^a^	$B\left(t\right)=\overset{n}{\underset{i=0}{\sum }}b_{i,n}\left(t\right)P_{i}$, *b*_*i*,*n*_(*t*) = *C*_*n*_^*i*^ *t* ^*i*^(1 − *t*)^*n*−*i*^	**p** = {**P**_0_, **P**_1_,…, **P**_*n*_} **P**_*i*_ = (*x*_*i*_, *y*_*i*_), *i* = 0,1,…, *n*

B-spline^b^	$S\left(t\right)=\overset{n}{\underset{i=0}{\sum }}P_{i}N_{i,k}\left(t\right)$ $N_{i,0}\left(t\right)=\left\{\begin{array}{cc} 1, & if\;\;t\in \left[u_{i},u_{i+1}\right]\\ 0, & otherwise \end{array}\right.$ $N_{i,k}\left(t\right)=\frac{t-u_{i}}{u_{i+k}-u_{i}}N_{i,k-1}\left(t\right)+\frac{u_{i+k+1}-t}{u_{i+k+1}-u_{i+1}}N_{i+1,k-1}\left(t\right)$ **U** = [*u*_0_, *u*_1_,…, *u*_*n*+*k*+1_], *u*_0_ = 0, *u*_*n*+*k*+1_ = 1	**p** = {**P**_0_, **P**_1_,…, **P**_*n*_} **P**_*i*_ = (*x*_*i*_, *y*_*i*_), *i* = 0,1,…, *n*

^a^Bernstein basis polynomial of Bézier curve is denoted by *b*_*i*,*n*_.

^b^B-spline is a piecewise polynomial function of degree *k*, which is defined by *n* + 1 control points and *n* + *k* + 2 knots **U**. Coefficient *N*_*i*,*k*_ is obtained by recurrence relation.

**Table 2:** Configurations for TST model verification and validation.

Geometry configurations								Photon source configurations			
	SAD	SCD	SDD	lh	dh	FS	*ρ*	Energy	*μ*_*a*_/*ρ*	FWHM	*α*_*B*_
Unit	cm	cm	cm	cm	cm	cm	g/cm^3^	MeV	cm^2^/g	cm	—
Value	100	46	33.9	8	7.8	40	19.3	1.5	0.05	0.2	15.8°

**Table 3:** Leaf end curve constraints.

Type	Bound constraints	Linear constraints
Circular arc^a^	$\frac{\text{lh}}{2}\leq R<+\infty$	$-R+d+\frac{\text{lh}}{2}\leq 0$
	$-\frac{\text{lh}}{2}\leq d\leq \frac{\text{lh}}{2}$	$-R-d+\frac{\text{lh}}{2}\leq 0$

Elliptical arc^b^	$a=\frac{\text{lh}}{2}$	$0<{\left(1-\frac{b^{2}}{a^{2}}\right)}^{1/2}<1$
	$0\leq b\leq \frac{\text{lh}}{2}$	

Bézier curve	$\left(x_{0},y_{0}\right)=\left(-\frac{\text{lh}}{2},0\right)$, $\left(x_{n},y_{n}\right)=\left(\frac{\text{lh}}{2},0\right)$	*y*_*i*_ + *y*_*i*+2_ − 2*y*_*i*+1_ ≤ 0, *i* = 0,1,…, *n* − 2
	$\left(-\frac{1}{2}+\frac{i-1}{n-1}\right)\text{lh}\leq x_{i}\leq \left(-\frac{1}{2}+\frac{i}{n-1}\right)\text{lh}$ $0\leq y_{i}\leq \frac{\text{lh}}{2},\;i=1,\ldots ,n-1$	

B-spline	$x_{i}=\left(-\frac{1}{2}+\frac{i}{n}\right)\text{lh}$, *i* = 0,1,…, *n*	*y*_*i*_ + *y*_*i*+2_ − 2*y*_*i*+1_ ≤ 0, *i* = 0,1,…, *n* − 2
	$0\leq y_{j}\leq \frac{\text{lh}}{2}$, *j* = 0,1,…, *n* − 1,*y*_*n*_ = 0	

^a^Leaf end is straight when *R* tends to infinity.

^b^The special case of elliptical arc is circular arc when *b* equals lh/2.

**Table 4:** Algorithmic efficiency comparison of three algorithms.

Algorithmic efficiency	Monte Carlo	Ray tracing	TST model
Number of leaf positions	17	17	17
Number of histories/line intersections^a^	10^9^	10^4^	2
Computation time^b^	33.15 h	8.45 min	0.33 s

^a^Number of histories or line intersections is calculated for a given leaf position.

^b^Timing is recorded on the same workstation without using parallel computing.

**Table 5:** Summary of optimal leaf ends and penumbral properties.

Leaf end curve	Design variables (cm)	Penumbra mean (cm)	Standard deviation (cm)
Circular arc	*R* = 16.213, *d* = −0.732	0.198	0.0130

Elliptical arc	*b* = 0.728	0.209	0.0207

Cubic Bézier	**P**_1_ = (−1.594, 1.077), **P**_2_ = (0.814, 0.748)	0.210	0.0005

B-spline^a^	**Y** = (0.401, 0.604, 0.699, 0.718, …, 0.671, 0.572, 0.422, 0.240)	0.197	0.0066

^a^ **Y** denotes the set of *y*-axis values of control points **P**_0_ to **P**_7_.

**Table 6:** Results of TST model based optimization and empirical method compared with actual leaf ends.

Type	Actual shape (cm)	Empirical method (cm)	TST model (cm)
Elekta Agility 160-leaf MLC^a^	*R* = 17	*R* = 20.9	*R* = 16.805, *d* = −1.153

Varian Millennium 120 MLC^b^	*R* = 8, *α*_*l*_ = 11.3°	*R* = 8	*R* = 12.354, *d* = −0.125

^a^SCD = 35.1 cm, leaf height = 9 cm [14].

^b^SCD = 51.02 cm, leaf height = 5.65 cm [15]. Angle *α*_*l*_ denotes the angle between line segment of piecewise leaf end curve and collimator rotation axis.

## Appendices

### A. Algorithm for Tangent Line Computation

Root finding approach for tangent line computation is illustrated in Figure 15.

Given an arbitrary point *D* on parametric leaf curve, *t*_*D*_ denotes the value of independent parameter *t* at point *D*. Vector **a** denotes tangent vector at point *D*, and **b** denotes vector of $\vec{DE}$. The tangent vector for point on distal side and proximal side is given by vector (0,1) and (0, −1), respectively. Point *D*_0_ locates where parameter *t* equals 0, with tangent vector of **a**_0_. Note that the angle between **a** and **b** is minimal at the point of tangency; iterative method is performed for finding the solution. Since the objective function is not convex, a piecewise function with unique global minimum is created, which is given as follows:

(A.1) $$\begin{array}{c} \min\alpha \left(\;t_{D}\;\right),\\ \alpha \left(\;t_{D}\;\right)=\left\{\begin{array}{cc} \arccos\frac{a\cdot b}{\left\|a\right\|\left\|b\right\|}, & t\geq 0\\ -\arccos\frac{a\cdot b}{\left\|a\right\|\left\|b\right\|}+2\alpha_{0}, & t<0, \end{array}\right.\\ \alpha_{0}=\arccos\frac{a_{0}\cdot b_{0}}{\left\|a_{0}\right\|\left\|b_{0}\right\|}. \end{array}$$

In case that the resulting *t*_*D*_ falls out of the range of [0,1], a switch statement is given as

(A.2) $$\begin{array}{c} t_{D}=\left\{\begin{array}{cc} 0, & t_{D}<0,\\ 1, & t_{D}>1. \end{array}\right. \end{array}$$

### B. Algorithm for Secant Line Computation

Root finding approach for secant line computation is illustrated in Figure 16.

Given an arbitrary point *B* on parametric curve, search for point *A*, satisfying the condition that secant point *A*, secant point *B*, and point *C*, defined by the equivalent source size, are collinear, while the length of chord equals the effective path length *l*. Root finding approach for secant line computation is given as finding the solution of a system of equations, which is written as

(B.1) d=l,cc=−dd,

where vector **c** denotes vector of $\vec{BC}$ and **d** denotes vector of $\vec{BA}$.

The first step is that for a given point *B* find the mapping point *A* on the parametric curve with condition of ‖**d**‖ = *l*. It should be noted that, for point *B*, there exist two solutions for the mapping condition on parametric curve. In our work, the solution of point *A* with inequality constraints of *t*_*A*_ < *t*_*B*_ is desired. A piecewise function for point mapping is given as follows:

(B.2) min⁡ΔltA,ΔltA=d−l,if  tA<tBd+l+ktA−tB, k∈R+,otherwise,

where penalty function *k*(*t*_*A*_ − *t*_*B*_) is introduced with penalty coefficient *k*.

The second step is solving the equation of collinearity. Note that the angle between **c** and **d** is maximal on the desired secant line; iterative method is performed for finding the solution. Since the objective function is not convex, a piecewise objective angle function with unique global minimum is written as follows:

(B.3) $$\begin{array}{c} \min-\beta \left(\;t_{B}\;\right),\\ \beta \left(\;t_{B}\;\right)=\left\{\begin{array}{cc} \arccos\frac{c\cdot d}{\left\|c\right\|\left\|d\right\|}, & t\geq 0\\ -\arccos\frac{c\cdot d}{\left\|c\right\|\left\|d\right\|}+2\beta_{0}, & t<0, \end{array}\right.\\ \beta_{0}=\arccos\frac{c_{0}\cdot d_{0}}{\left\|c_{0}\right\|\left\|d_{0}\right\|}. \end{array}$$
